# Autophagy inhibition enhances Matrine derivative MASM induced apoptosis in cancer cells via a mechanism involving reactive oxygen species-mediated PI3K/Akt/mTOR and Erk/p38 signaling

**DOI:** 10.1186/s12885-019-6199-7

**Published:** 2019-10-15

**Authors:** Yuming Zou, Melika Sarem, Shengnan Xiang, Honggang Hu, Weidong Xu, V. Prasad Shastri

**Affiliations:** 1 Institute for Macromolecular Chemistry, University of Freiburg, 79104 Freiburg, Germany; 2 Department of Orthopaedics, Department of Orthopaedics, People’s Hospital of Wuhan University, Wuhan, 430060 Hubei Province People’s Republic of China; 3 Department of Orthopaedics, Changhai hospital, Second Military Medical University, Shanghai, 200433 People’s Republic of China; 4 Department of Orthopaedics, the 904th Hospital of Joint Logistic Support Force, Chinese People’s Liberation Army, Wuxi, Jiangsu Province People’s Republic of China; 5 BIOSS Centre for Biological Signalling Studies, University of Freiburg, 79104 Freiburg, Germany; 6 Department of Organic Chemistry, School of Pharmacy, Second Military Medical University, Shanghai, 200433 People’s Republic of China

**Keywords:** Matrine, Matrine derivate, Autophagy, Apoptosis, Anti-cancer, Reactive oxygen species

## Abstract

**Background:**

In the quest for new anti-cancer drugs, the drug discovery process has shifted to screening of active ingredients in traditional eastern medicine. Matrine is an active alkaloid isolated from plants of the Sophora genus used in traditional Chinese herbal medicine that exhibits a wide spectrum of biological properties and has a potential as an anti-proliferative agent. In this study, we investigated the anticancer property of MASM, ([(6aS, 10S, 11aR, 11bR, 11cS)210-Methylamino-dodecahydro-3a, 7a-diaza-benzo (de)anthracene-8-thione]), a potent derivative of matrine.

**Methods:**

Four epithelial cancer cell lines representing the dominant cancers, namely: A549 (non-small-cell lung cancer cell line), MCF-7 and MDA-MB-231 (breast cancer cell lines), and Hela (cervical cancer cell line) were employed, and the mechanistic underpinning of MASM-induced apoptosis was investigated using flow cytometry, western blot and immunofluorescence.

**Results:**

MASM, induced apoptosis via caspase 3 dependent and independent pathways, and autophagy in all the four cancer cell lines, but post-EMT (epithelial mesenchymal transition) cells showed greater sensitivity to MASM. Scavenging reactive oxygen species using N-acetylcysteine rescued all cancer cell lines from apoptosis and autophagy. Mechanistic analysis revealed that MASM induced autophagy involves inhibition of Akt signaling and the activation of Erk and p38 signaling, and inhibition of autophagy further enhanced the apoptosis induced by MASM.

**Conclusions:**

These results indicate that MASM possesses potency against cancer cells and modulating autophagy during MASM administration could be used to further enhance its therapeutic effects.

## Background

Cancer is the second leading cause of death worldwide. In 2015, there were 17.5 million incidents of cancer and 8.7 million cancer related deaths (15.7% of deaths) [1, 2]. Overall, breast cancer, TBL (tracheal, bronchus, and lung) cancer, colorectal cancer, prostate cancer, stomach cancer, liver cancer, non-Hodgkin lymphoma, leukemia, bladder cancer and cervical cancer were the top 10 most common incident cancers in both sexes [1]. In addition to surgery and radiotherapy, chemotherapy remains the major option for cancer therapy, especially for metastatic cancers [3]. However, the side-effects of chemotherapy and development of chemo-resistance in cancer cells are persistent challenges. Thus, developing novel therapeutic agents and enhancing the therapeutic efficacy of anticancer drugs carries substantial clinical value.

Autophagy is an evolutionarily conserved lysosomal degradation pathway that maintains intracellular homeostasis, in baseline conditions and in the context of adaptive responses to stress, by eliminating damaged organelles and protein aggregates [4]. Autophagy plays negative and positive roles in cancer therapy, primarily protective to cancer cells as a mechanism of chemoresistance but can also lead to type II cell death (autophagic cell death) [5, 6]. So, it is imperative to unveil the role of autophagy in anticancer therapy before targeting it as part of a combination therapy with anticancer therapeutic agents, which could provide the opportunity for encapsulation of MASM in polymeric or lipid-based nanoparticles and vehicles for targeted therapeutics [7–9].

Matrine, is an active alkaloid compound that is isolated from plants of the *Sophora* genus used in traditional Chinese herbal medicine*.* It possesses a variety of pharmacological properties [10], such as anticancer [5, 11–13], anti-inflammatory [14–18], antiviral [19–21], and anti-fibrotic activities [22]. However, matrine has low therapeutic efficacy, thus a series of matrine derivatives have been designed and synthesized, among them MASM [(6aS, 10S, 11aR, 11bR, 11cS)210-Methylamino-dodecahydro-3a, 7a-diaza-benzo (de)anthracene-8-thione], which exhibits greater anti-inflammatory property in vitro [23]. There are also studies showing that the matrine derivative MASM also has immunomodulatory properties [24], prevents fibrosis [25], is anti-osteoporotic [26], offers radioprotection after lethal full body radiation [27], and is anti-inflammatory [28]. Considering the diverse pharmacological activity of MASM the exact mechanism by which it can function as an anticancer agent needs further elucidation.

In this study, we evaluated the anticancer properties of MASM on A549 (non-small cell lung cancer cell line), MCF-7 and MDA-MB-231(breast cancer cell lines), and Hela (cervical cancer cell line) and the associated mechanisms. Our findings demonstrate that MASM induces apoptosis and autophagy in all cancer lines. In addition, the inhibition of autophagy results in enhancement of MASM-induced apoptosis through reactive oxygen species (ROS)-mediated PI3K/Akt/mTOR, Erk and p38 signaling pathway.

## Methods

### Reagents

MASM [(6aS,10S,11aR,11bR,11cS)-10-methylamino-dodecahydro-3a,7a-diazabenzo (de)anthracene-8-thione] (purity > 99%) was synthesized and characterized as reported earlier [23]. Chloroquine (Sigma, Germany), N-Acetyl-L-cysteine (NAC, Sigma, Germany) were dissolved in phosphate buffered saline (PBS). LY294002 (Invivogen, Germany), Wortmaninn (Invivogen, Germany), PD184352 (Sigma, Germany), SB230580 (adooq biosciences, USA) were dissolved in dimethyl sulfoxide (DMSO).

### Cell culture

All epithelial cancer cell lines were provided by the BIOSS (Centre for Biological Signalling Studies, University of Freiburg) Toolbox and were genotyped and verified by Labor für DNA Analytik (Freiburg, Germany). All cells were tested for mycoplasma at the BIOSS Toolbox and were used between 3 and 5 passages after thawing. A549 and MDA-MB-231 were cultured with Dulbecco’s modified Eagle’s medium (DMEM, Gibco Invitrogen) supplemented with 10% fetal bovine serum (FBS), 1% penicillin/streptomycin. While MCF-7 and Hela cells were cultured with RPMI Media 1640 supplemented with 10% FBS, 1% penicillin/streptomycin (all reagents from Invitrogen). Cells were cultured in humidified atmosphere in a 37 °C incubator at 5% CO2.

### MTT assay

Viability of cells were assessed by 3-(4,5-Dimethylthiazol-2-yl)-2,5-diphenyltetrazolium bromide (MTT) assays. Cells were seeded in a 96 well plates (A549 7000 cells/well, MCF-710000 cells/well, MDA-MB-2317000 cells/well, Hela 5000 cells/well) and after 12 h treated with MASM at different concentrations ranging from 0 to 120 μg/ml for 8, 16, and 24 h. Supernatants were removed and 100 μl of MTT solution (5 mg/mL) was added at the end of incubation, and three hours later the absorbance value at 570 nm was measured on a microtiter plate reader (Bio-Tek instrument, USA). All MTT assays were performed in triplicates and minimum of three independent experiments. The metabolic activity of cells was calculated according to the formula: 100% × (experimental -blank absorbance value) / (control-blank absorbance value).

### Lactate dehydrogenase assay

The effect of MASM on the integrity of plasma cell membrane was accessed by LDH-Cytotoxicity Assay Kit II (Abcam, Germany). LDH is released by the cells into the culture supernatant in response to damage to cell membrane integrity and can be used as an indicator of cytotoxicity. After incubation with MASM 60 μg/ml for various time periods (2,4,6,8,12,24 h) the supernatants were collected and centrifuged at 1000 x g for 5 min to remove the sediments and then used for LDH assay following the protocol provided by manufacture. The absorbance value at 450 nm was measured on a microtiter plate reader (Bio-Tek instrument, USA).

### Flow cytometry analysis for apoptosis

Cells were seeded and allowed to attach overnight and then incubated with different concentrations of MASM for 24 h. Apoptosis was detected using Annexin V-FITC/PI Apoptosis Detection kit (BD biosciences, San Jose, California, USA) and analyzed by flow cytometry with FACS Gallios flow cytometer (Beckman coulter).

### Western blot analysis

Cell extracts were prepared by lysing the cells in RIPA buffer with 1% proteinase and 1% phosphate inhibitors. Proteins were boiled with Laemmli buffer for 5 min at 95 °C, and the gel was loaded at a concentration of about 20~30 μg of protein/loading well and electrophoretically separated using sodium dodecyl sulfate-polyacrylamide gel electrophoresis (SDS-PAGE) with 10% or 12% gel and then transferred to a 0.22 μm polyvinylidenefluoride (PVDF) membrane. After blocking with 5%(w/v) bovine serum album (BSA) in TBS-T buffer (20 mM Tris (pH 7.4), 150 mM NaCl, and 0.1% Tween 20), membranes were incubated with primary and then peroxidase-conjugated secondary antibodies. The intensities of bands were visualized with chemiluminescence solution (Thermo Scientific, Germany) through a digital gel-imaging system (PeqLab Fusion FX7, PeqLab, Germany).

### Immunofluorescence for LC-3

The LC-3 expression levels were determined using an immunofluorescence analysis. Cells were seeded in an 8-well Tissue Culture Chambers (Sarstedt AG & Co, Germany). After treatment with MASM 60 μg/ml with or without 50 μM Chloroquine for 8 h, the cells were fixed with 4% (v/v) paraformaldehyde (in PBS) for 10 min. After fixation, the cells were permeabilized with cold methanol for 10 min at − 20 °C and blocked with 2% (v/v) fetal bovine serum (FBS) and 1% (v/v) Goat Serum in PBS for 1 h at room temperature. After blocking, the cells were incubated with primary LC-3 antibody (1:100 diluted in blocking buffer) at − 4 °C overnight and then incubated with FITC-conjugated anti-rabbit IgG secondary antibody at room temperature for 1 h. Coverslips are mounted with DAPI (Invitrogen) to stain the nuclei. Samples were visualized using Cell Observer Z1 (Carl Zeiss Microscope, Germany) and images acquired were analyzed using Zeiss Zen Blue software suite.

### Statistical analysis

Data are presented as mean ± standard deviation (SD) of *n* ≥ 3 and analyzed using GraphPad Prism 6 (USA). Analysis of variance (ANOVA) was used to analyze differences between groups with the threshold significance level set at *P* < 0.05.

## Results

### Effect of MASM on cell viability and cellular toxicity

**Fig. 1:**
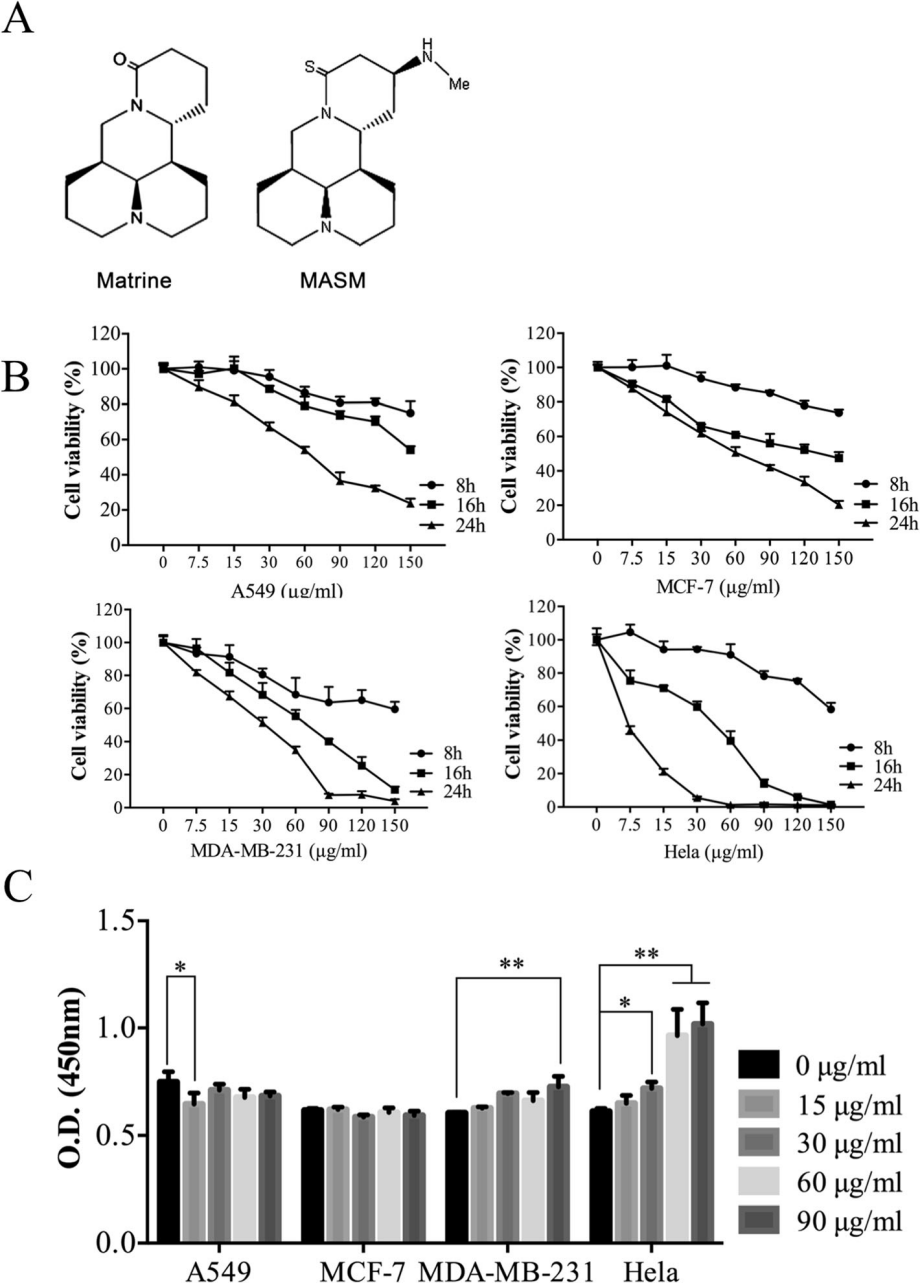
MASM induced a dose- and time-dependent inhibitory effect on cell viability. **a** Chemical structure of matrine and its derivate MASM; **b** Viability of cells follwoing exposure to different concentrations of MASM for 8 h,12 h and 24 h. Cell viability was determined using MTT assay; **c** The effect of MASM treatment for 24 h on LDH release. Data are representative of at least three independent experiments

The chemical structure of matrine and MASM is shown in Fig. 1a. To investigate the effect of MASM on cell viability in cancer cells, cells were treated with different concentrations (from 0 to 150 μg/ml of MASM for various time points (8 h, 16 h and 24 h). As shown in Fig. 1b, MASM induced a dose- and time-dependent inhibitory effect on the viability of A549, MCF-7, MDA-MB-231 and Hela cells. However, this dose-dependent toxicity manifested itself at lower doses at 24 h in MDA-MB-231 and HeLa cells. In order to investigate the kinetics of MASM cytotoxicity, the effect of MASM on LDH release as a function of dosage (0, 15, 30, 60, 90 μg/ml) and time (2 h, 4 h, 6 h, 8 h, 12 h and 24 h) was measured. While there was no difference in LDH release in A549 and MCF-7 for the various MASM concentrations studied (from 15 μg/ml to 90 μg/ml) for up to 24 h (Additional file 1: Figure S1); in MDA-MB-231 and Hela cells, with an increase in the concentration of MASM to 90 μg/ml, there was a statistically significant increase in LDH release over 24 h in both MDA-MB-231 and HeLa cells (Fig. 1c). These results indicated that while MASM has no acute cytotoxicity, long-term exposure appeared to be more toxic to post-EMT cells lines.

### MASM induces apoptosis in cancer cells

**Fig. 2:**
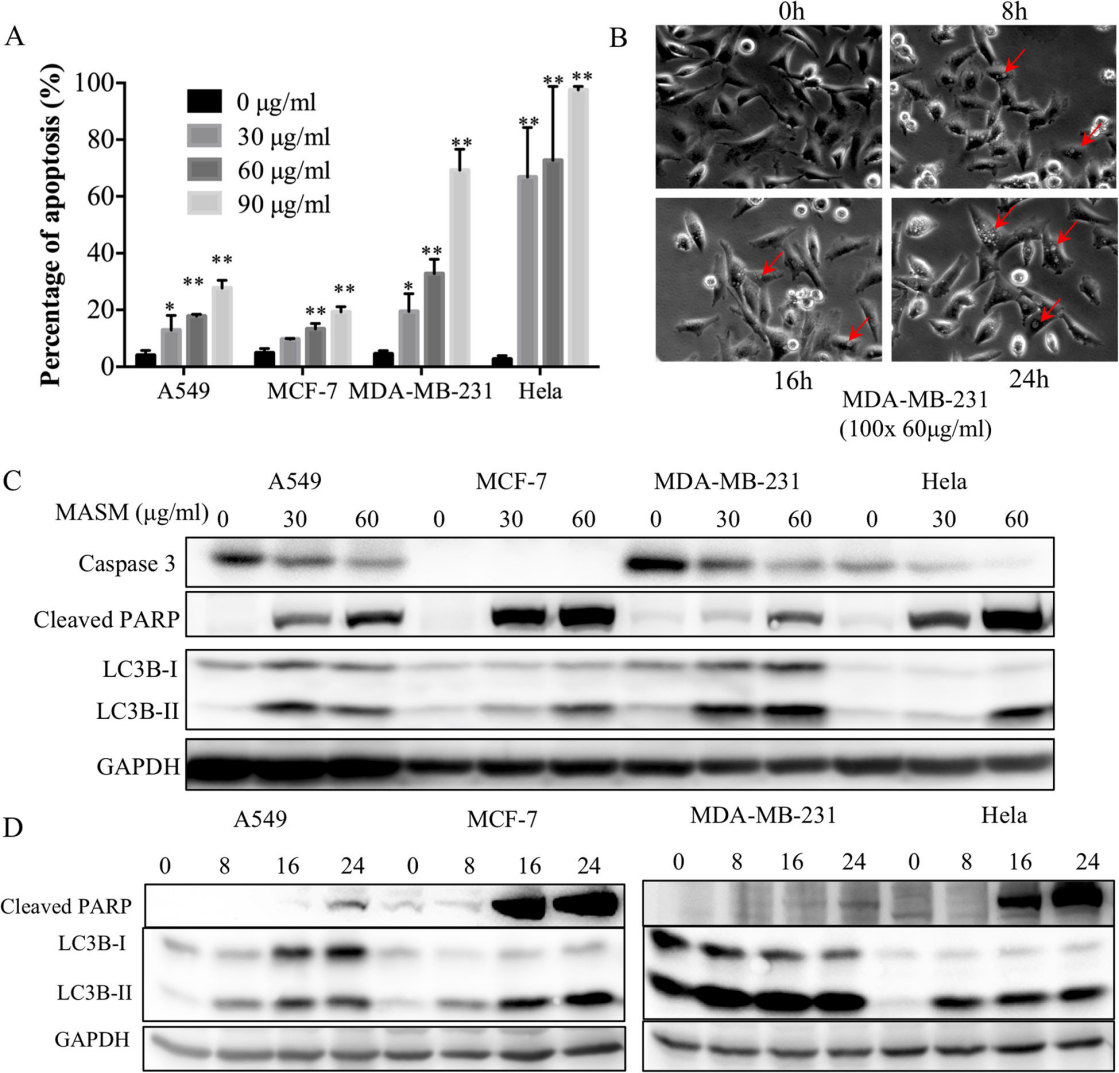
Matrine derivate MASM induces apoptosis and autophagy in A549, MCF-7, MDA-MB-231 and Hela. **a** Effect of MASM on cell apoptosis. Apoptosis in cells treated with different concentrations of MASM for 24 h assessed using Annexin V-FITC/PI double staining and quantified by flow cytometry. Bars show the mean ± SD from three independent experiments; **b** Cytoplasm vacuoles observed under light microscope; **c** Cells were treated with 0, 30, 60 μg/ml of MASM for 24 h, western blotting was performed to detect expression of Caspase 3, Cleaved PARP and LC3B; **d** Cells were treated with 60 μg/ml of MASM, western blotting was performed to detect expression of Cleaved PARP and LC3 at 0 h, 8 h, 16 h and 24 h. Data were representative of three independent experiments

To investigated whether MASM inhibits the proliferation of the cancer cells via induction of apoptosis, cells were seeded and cultured overnight and then treated with increasing concentrations of MASM for 24 h, and then double stained using Annexin V/PI and characterized using flow cytometry. As shown in Additional file 1: Figure S2 (flow cytometry charts) and Fig. 2a (quantitative analysis), MASM induced apoptosis in A549, MCF-7, MDA-MB-231 and Hela in a dose-dependent manner. In addition, at the same dosage MASM induced more apoptosis in MDA-MB-231 and Hela cells than in A549 and MCF-7. These results were consistent with what was found in MTT and LDH assays, that post-EMT cells are more susceptible to MASM. In addition, during the course of MASM treatment, we noticed under the light microscope that there were cytoplasm vacuoles accumulated in the cells, which was a morphological feature of autophagy (Fig. 2b, MASM 60 μg/ml, MDA-MB-231 as an example).

Caspases are crucial mediators of apoptosis, among them, caspase 3 is a key “executioner” of the apoptotic machinery and it is cleaved into two subunits when the cells undergo apoptosis [29]. Poly (ADP-ribose) polymerase (PARP) is one of the substrates of caspases and its cleavage by caspases is considered a hallmark of apoptosis [30]. MASM induced a dose- and time-dependent increase in the cleavage of PARP in A549, MCF-7, MDA-MB-231 and Hela cells, which was consistent with the result obtained using Annexin V/PI double staining. Furthermore, a dose-dependently decrease in expression of pro-caspase 3 were found in A549, MDA-MB-231 and Hela. However, as MCF-7 is caspase 3 deficient, there was no pro-caspase 3 expression in the lysates of these cells (Fig. 2c). Since, the executioner caspases include 6 and 7 in addition to the major executioner caspase, caspase 3, and MCF-7 are deficient in caspase 3, this implies that MASM can induce apoptosis through both a caspase 3-dependent and caspase 3-independent pathway in cancer cells.

### MASM induces autophagy through PI3K/Akt/mTOR signaling pathway

**Fig. 3:**
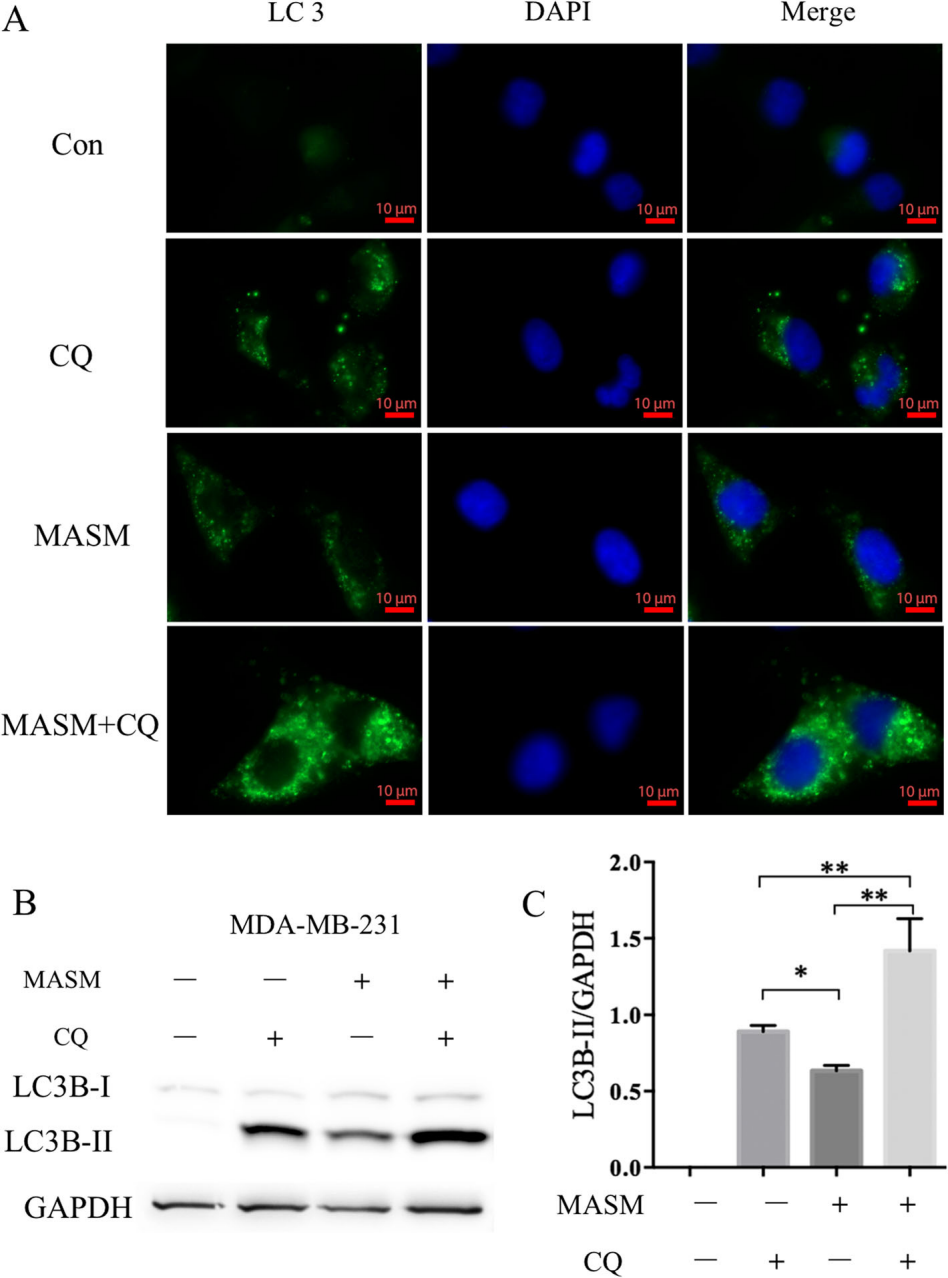
MASM increased autophagic flux in MDA-MB-231. **a** MDA-MB-231 were treated with MASM (60 μg/ml) with or without CQ (10 μM), and the immunofluorescent was adopted to detect the LC3 puncta within the cells; **b** MDA-MB-231 were treated with MASM (60 μg/ml) with or without CQ, and western blot was adopted to detect the expression level of LC3B-II; **c** Quantitative analysis of bands intensities of LC3B-II by Image J. Data are representative of three independent experiments. *P < 0.05, **P < 0.01

The conversion of microtubule-associated protein light chain 3 (LC3) proteins (LC3-I to LC3-II) are involved in the formation of autophagosomes, and is now widely applied in monitoring autophagy. Also, the amount of LC3-II is clearly correlated with the amount of autophagosomes, and thus serves as a relatively accurate marker of autophagy [31]. As shown in Fig. 2c, MASM also induced a dose-dependent accumulation of LC3II in the four cancer cell lines under investigation. The time-course experiment revealed that MASM (60 μg/ml) treatment markedly induced LC3-II accumulation as early as 8 h (Fig. 2d). However, the accumulation of LC3-II is only indicative of an increase in the amount of autophagosome and this could be either due to increased autophagic activation or a blockage of subsequent breakdown of autophagosome [32]. Chloroquine (CQ) is known to prevent the acidification of the lysosome thereby blocking autophagosome-lysosome fusion and autophagy at the late stages [33]. CQ is therefore widely used as an autophagic inhibitor [34]. To further unveil the influence of MASM to autophagic flux, MDA-MB-231cells were treated with MASM (60 μg/ml) with or without CQ (10 μM) for 8 h and cells were immunostained for LC3-II and lysates analyzed by western blotting. Cells treated in presence of CQ showed an increase in the punctate staining for LC3-II (Fig. 3a) and this was concurrent with the increase in LC3-II expression at the protein level, indicating that MASM induced autophagy and increased the autophagic flux in MDA-MB-231(Fig. 3b and c).

**Fig. 4:**
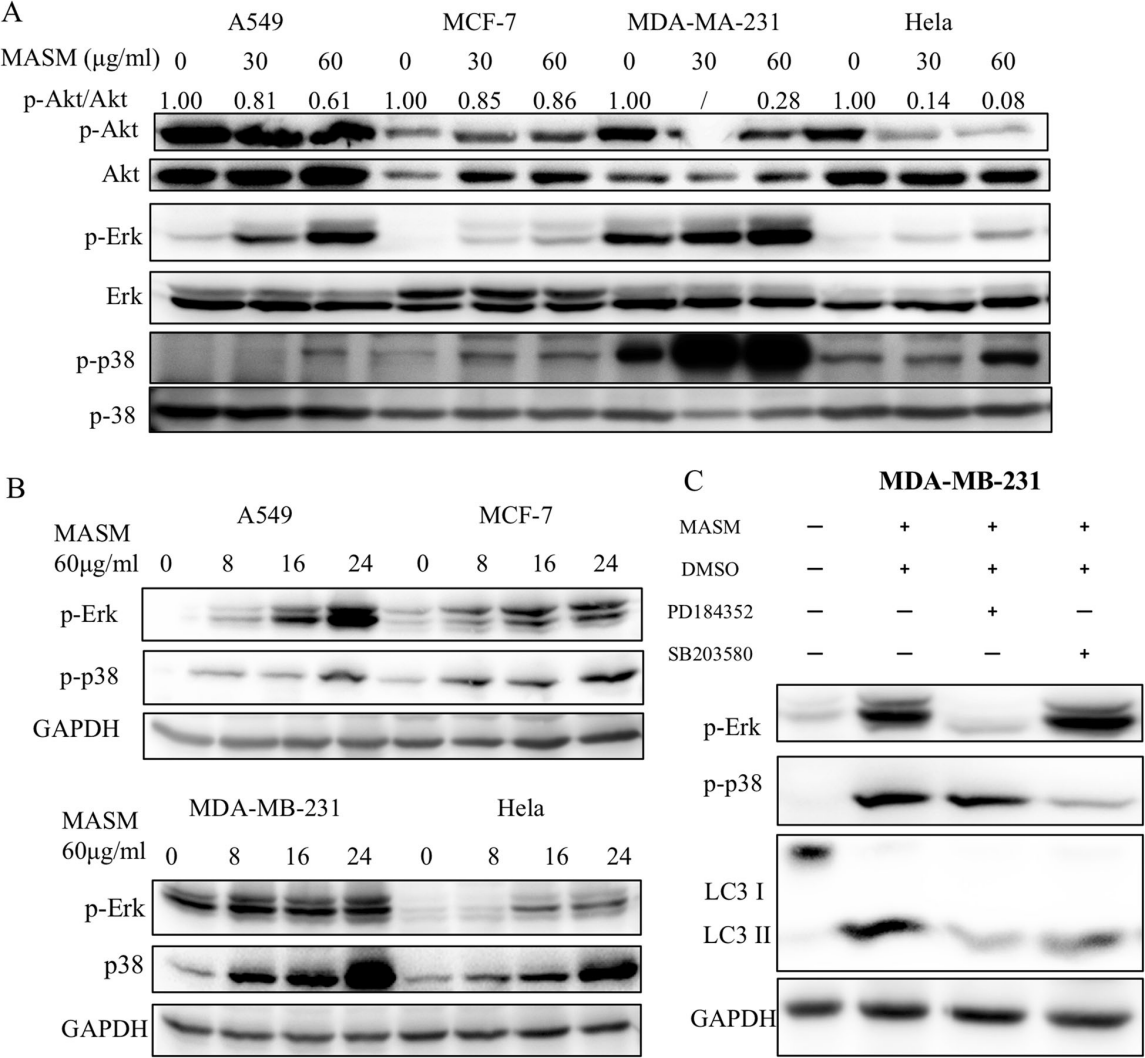
MASM induces autophagy through PI3K/Akt/mTOR, Erk1/2 and p38 signaling pathway. **a** Indicated cancer cell lines were treated with different concentrations of MASM (0, 30, 60 μg/ml) for 24 h, then expression levels of p-Erk1/2, Erk1/2, p-p38, and p38 were carried out by western blot. **b** Indicated cancer cell lines were treated with 60 μg/ml MASM for different times (0,8,16,24 h) and the relative expression levels of p-Erk1/2, p-p38 compared to GAPDH. **c** Expression of p-Erk, p-38 and LC3B-II in MDA-MB-231 treated with MASM alone or co-treatment with Erk1/2 inhibitor PD184352 (2 μM) or p38 inhibitor SB203580 (10 μM). Data were representative of three independent experiments

It has been shown that activation of class I PI3K negatively regulates autophagy indirectly through the well-established PI3K/Akt/mTOR signaling pathway [35, 36]. Akt is a central player in PI3K/Akt/mTOR signal transduction as its phosphorylation can lead to the activation and signaling through the mTORC1 (mechanistic target of rapamycin complex 1). Thus, to see if autophagy induced by MASM occurs through PI3K/Akt/mTOR signaling pathway we investigated the effects of MASM on the activation of Akt [37]. It was found that MASM markedly inhibits the phosphorylation of Akt in a dose dependent manner in all the four cancer cell lines investigated (Fig. 4a).

### MASM induced autophagy also involves Erk1/2 and p38 signaling pathway

It has been reported that Erk1/2 and p38 are also involved in the regulation of autophagy [37–39]. To investigate the effects of MASM treatment on the signaling of Erk1/2 and p38, the expression levels of p-Erk1/2 and p-p38 were examined by western blot. As shown in Fig. 4, MASM treatment induced a dosage- and time-dependent increase in the phosphorylation of Erk1/2 and p38 (Fig. 4b). To further determine the role of Erk1/2 and p38 activation in MASM induced autophagy, studies were carried out in presence of specific inhibitors of Erk1/2 (PD184352) and p38 (SB203580). Pretreatment with PD184352 (2 μM) significantly attenuated the MASM induced activation of Erk1/2 and concomitantly the accumulation of LC3-II. Similar outcomes were observed with p38 inhibitor SB203580 (10 μM) as well, indicating that the activation of Erk1/2 and p38 played a role in MASM induced autophagy.

### Autophagy inhibition enhances apoptosis induced by MASM

**Fig. 5:**
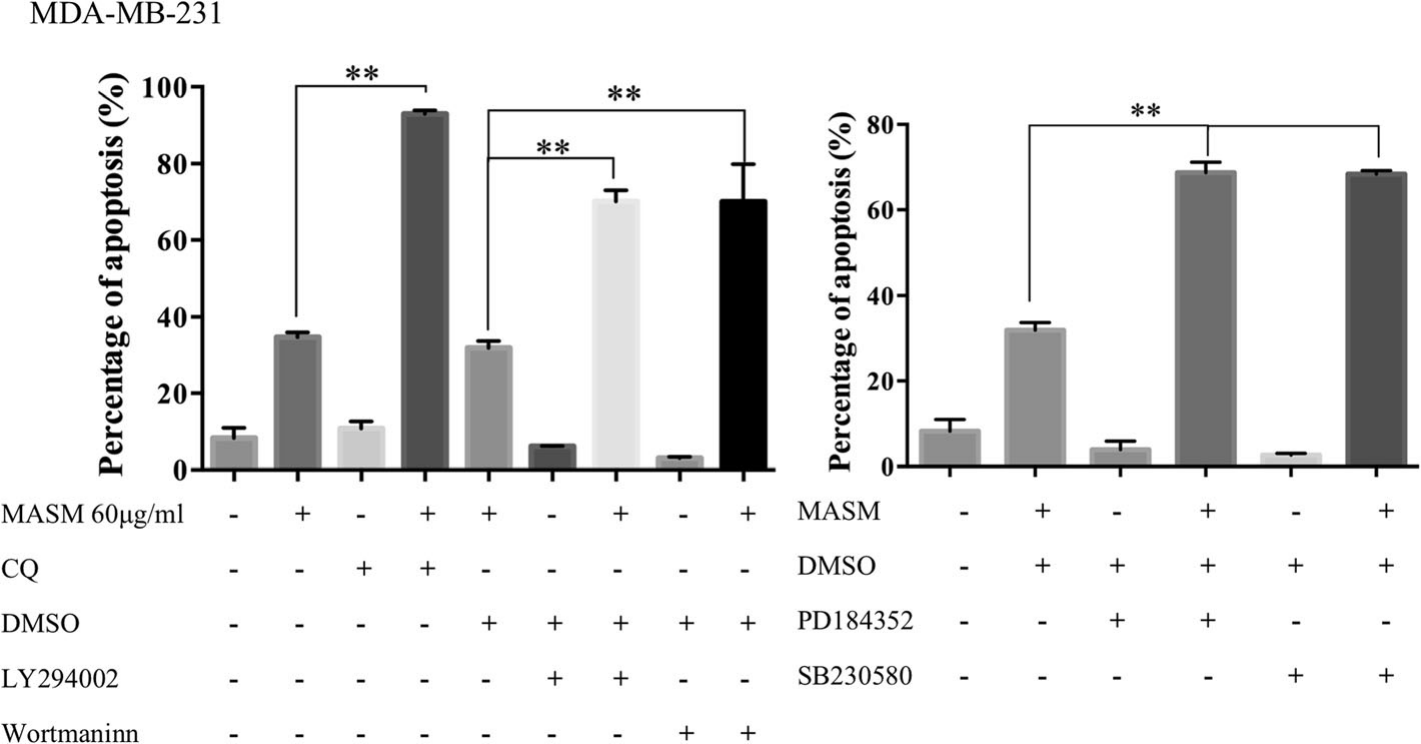
Inhibition of MASM induced autophagy enhanced apoptosis in MDA-MB-231. A. The apoptosis in MDA-MB-231 induced by MASM (60 μg/ml) in the presence or absence of autophagy inhibitor CQ (10 μM), LY294002 (10 μM), or Wortmaninn (1 μM) was analyzed by flow cytometry using Annexin V/PI double staining. B. The apoptosis in MDA-MB-231 induced by MASM in the presence or absence of PD184352 (2 μM) or SB203580 (10 μM), was analyzed by flow cytometry using Annexin V/PI double staining. Results are shown as the mean ± SD of 3 independent experiments. *P < 0.05, **P < 0.01

Having established that MASM induced both apoptosis and autophagy in cancer cells, we set about to further unravel the relationship between apoptosis and autophagy in this paradigm. In addition to late stage autophagic inhibitor CQ, wortmannin and LY294002 can effectively block the early stage of autophagy by inhibiting class III PI3K (Vps34), which plays an important role in mediating autophagosome formation [40, 41]. Since we have shown that MASM induced autophagy occurs in part through the activation of Erk1/2 and p38, we wondered if inhibiting autophagy through Erk1/2 and p38 inhibition could influence the apoptosis induced by MASM. The results shown that treatment of CQ (late stage autophagic inhibitor) or Wortmannin (early stage autophagic inhibitor) or LY294002 (early stage autophagic inhibitor) or PD184352 (Erk1/2 inhibitor) or SB203580 (p38 inhibitor) alone did not affect apoptosis, while combined treatment with MASM significantly increased the percentage of apoptotic cell death in MDA-MB-231 in comparison to MASM alone (Fig. 5). Similar findings were also made in A549, MCF-7 and Hela, suggesting that the activation of Akt, Erk1/2 and p38 contributed to MASM induced autophagy, and the inhibition of autophagy could enhance the apoptotic cell death induced by MASM.

### MASM induces apoptosis and autophagy through ROS generation

**Fig. 6:**
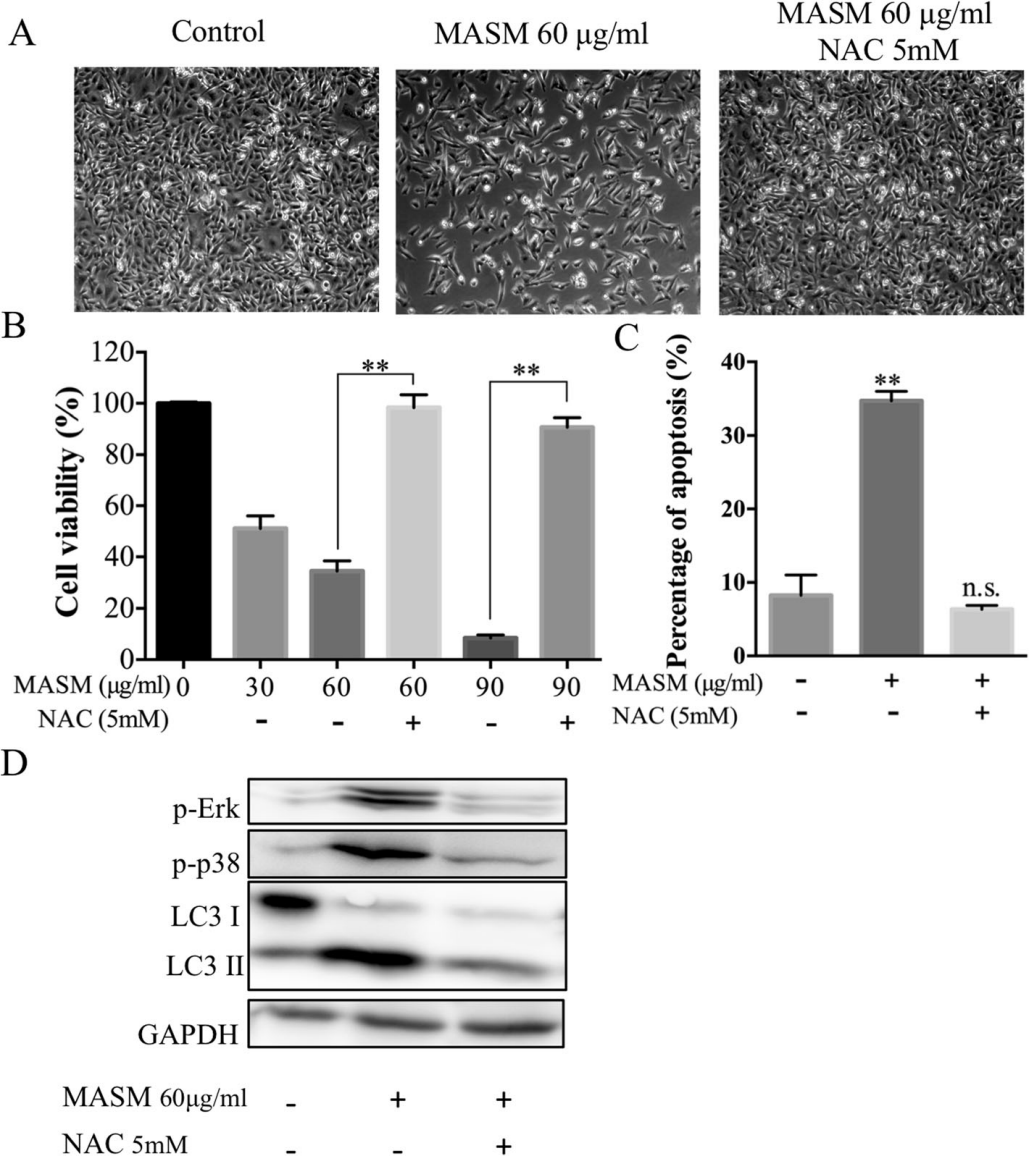
ROS scavenger NAC reversed the effects of MASM in MDA-MB-231. Administration of NAC (5 mM) with MASM could rescue the decrease in cell number as observed under a light microscope (**a**), cell viability as determined by MTT assay (**b**), rescue of cells from apoptosis as detected by Annexin V/PI double staining (**c**) and western blot showing the inhibitory effect of NAC on the MASM-induced activation of Erk1/2 and p38 and the accumulation of LC3-II (**d**). Bar graphs represent the mean ± SD of 3 independent experiments. *P < 0.05, ** *P* < 0.01

**Fig. 7:**
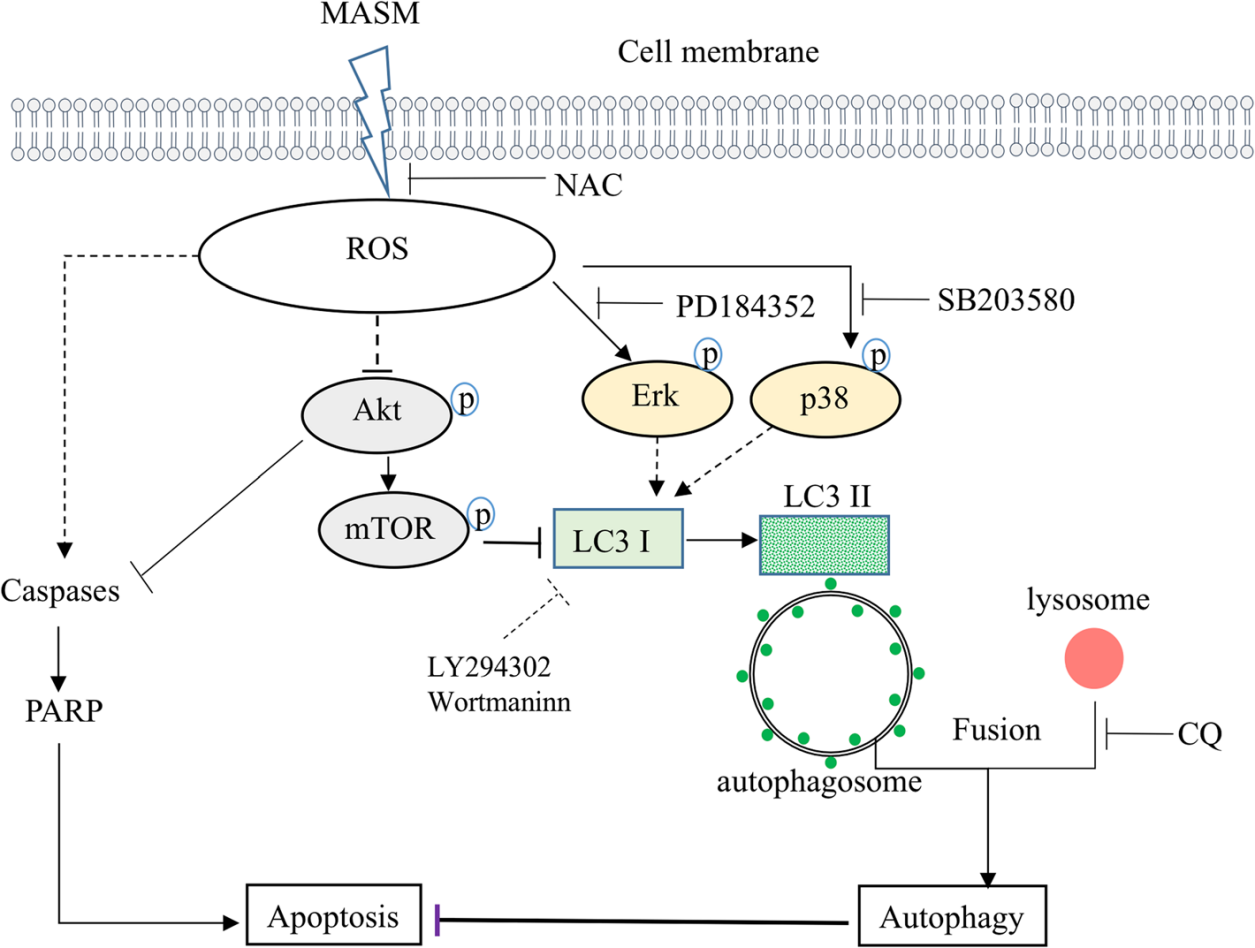
Schematic representation of the mechanism of MASM induced apoptosis and autophagy in cancer cells

Having confirmed the MASM induces apoptosis and autophagy in cancer cell lines, and that the inhibition of autophagy could enhance the apoptosis induced by MASM; we next investigated the underlying upstream molecular mechanisms leading to apoptosis and autophagy by MASM. Studies have reported that ROS generation plays a major role in several signaling pathways, and elevated ROS in cancer cells induces apoptosis or autophagy in response to chemotherapy-induced cellular stress [42–44]. A number of anticancer drugs have been found to exert their effects through activation of induced apoptosis or autophagy through ROS. Using MDA-MB-231 as an example, we therefore studied the apoptotic effects in presence of N-acetylcysteine (NAC), which is a scavenger of ROS [45, 46]. Scavenging ROS generation with NAC markedly rescued cell numbers as assessed by light microscopy (Fig. 6a) and MTT assay (Fig. 6b). These findings were also confirmed by flow cytometry (Fig. 6c). Western blot analysis showed that the activation of Erk1/2 and p38 by MASM and the accumulation of LC3-II were also inhibited by NAC (Fig. 6d). These findings in sum implicate ROS as the upstream molecular master regulator of MASM-induced apoptosis and autophagy. A mechanism of MASM-induced apoptosis and autophagy in cancer cells through ROS generation is depicted in Fig. 7.

## Discussion

Previous studies have reported that matrine, an active alkaloid compound isolated from *Sophora* genus plants, has a wide spectrum of pharmacological activities [10, 12]. However, the need for high dosage due to its low therapeutic efficiency have hampered its clinical exploitation. In this study, MASM, a synthetic derivative of matrine was studied for its anticancer properties in a panel of epithelial tumor cell lines. Our studies show that MASM can induce apoptosis in a dose-dependent manner through caspase 3-dependent manner (in A549, MDA-MB-213 and Hela) and caspase 3-independent manner (in MCF-7, which is caspase 3 deficient). Since pan-caspase inhibitor Z-VAD-FMK only partially rescues apoptosis induced by MASM in MDA-MB-231 (Additional file 1: Figure S3), one can conclude that MASM induces apoptosis via both caspase dependent and independent mechanisms. Several studies have investigated the anticancer activity of matrine. Lu et al. had reported an induction of ~ 25% apoptosis in A549 after 48 h exposure to matrine [47], and Wang et el., have shown that long-term (48 h) exposure of matrine at high doses (2000 μg/ml) can induce moderate (15%) apoptosis in cisplatin-resistance A549 [48]. Other studies have shown that matrine at a dose of 100 μg/ml matrine for 48 h can diminish proliferation of cervical cancer cells by reducing the expression of matrix metalloproteinases through suppression of p38 signaling pathway [49]. Other studies have reported apoptosis ranging from 20 to 25% in cancer cells after exposure to high doses of matrine (250 μg ~ 1000 μg/ml) for extended durations of 72 h [50, 51]. In comparison, MASM at a relatively modest dose of 90 μg/ml over 24 h induced appreciable apoptosis all in all cancer lines investigated here (~ 28% in A549, ~ 19% in MCF-7, ~ 69% in MDA-MB-231, and ~ 97% in Hela). Collectively this implies that the matrine derivate MASM may possess superior anti-proliferative properties in comparison to the parent alkaloid - matrine.

Autophagy, a self-degradation process that degrades cellular proteins and organelles during cellular stress not only prevents the toxic accumulation of damaged components but also recycles the degraded components, thus maintaining the cellular homeostasis. MASM induced an increase in LC3-II expression which is indicative of accumulation of autophagosomes within the cells. This increase in accumulation of autophagosome could be due to either increased autophagic flux or a blockade of autophagic flux. Studies in presence of late stage autophagic inhibitor CQ confirmed that this was due to increased autophagic flux in cells as both the punctate distribution of LC3-II and expression of LC3-II protein level was increased in the presence of CQ, in comparison with treatment with MASM or CQ alone. Furthermore, since MDA-MB-231 treated with MASM and CQ showed a further reduction in cell viability over cells treated with MASM alone (Additional file 1: Figure S4) this suggests a possible synergistic role for autophagy in MASM-induced apoptosis.

Previous studies have reported that MASM could inhibit PI3K/Akt signaling [26, 28] and here, too, MASM treatment was shown to significantly inhibit the expression of p-Akt in cancer cells. Since activation of Akt can lead to phosphorylation of mTOR, a negative regulator of autophagy, MASM-induced autophagy might involve PI3K/Akt/mTOR signaling pathway. However, contrary to the results of previous reports, which shown that MASM could inhibit the activation of MAPK signaling in LPS-induced RAW264.7 cells [28], murine bone-marrow dendritic cells [24], and RANKL/M-CSF induced osteoclastogenesis [26], we observed that the expression of Erk1/2 and p38 increased concomitantly with LC3-II, and furthermore, inhibiting the activation of Erk1/2 by PD184352 or p38 by SB203580 inhibited MASM induced autophagy. These data support the conclusion that Erk and p38 signaling pathways also play a role in MASM induced autophagy.

ROS are normal products of cellular metabolism. However, elevated ROS levels in cancer cells are correlated with apoptosis or autophagy in response to chemotherapy-induced cellular oxidative stress [42–44, 52]. Scavenging ROS with NAC rescued cancer cells from apoptosis and autophagy, indicating ROS production is an upstream regulator of MASM induced apoptosis and autophagy.

The role of autophagy in cancer is rather complex. During chemotherapy, autophagy may help cancer cells survive through the drug induced cellular stress by degradation of damaged mitochondria and toxic accumulation of damaged components, and maintaining metabolic homeostasis, thus leading to therapeutic resistance [5]. It has been found that both chemo and radiation therapy have an effect on autophagy. Thus, targeting autophagy in cancer therapy may help to improve the therapeutic efficiency of anticancer agents. CQ is an FDA approved drug for prophylactic treatment of malaria. Currently, there were a number of clinical trials utilizing CQ alone or in combination with other chemotherapies for the treatment of cancer, and the results indicated that CQ may not mediate therapeutic effects by itself but through enhancement of the therapeutic efficiency of anticancer drugs. As MASM induced autophagy and increased autophagic flux within the cancer cells through PI3K/Akt/mTOR, Erk1/2 and p38 signaling, one strategy to exploit MASM in treatment of cancer could involve co-delivery of late stage autophagy inhibitor CQ with inhibitors specific to either PI3K or p38 signaling pathways. Additionally, since MASM exhibits no acute toxicity in healthy human cells such as human pulmonary microvascular endothelial cells (Additional file 1: Figure S5) it warrants further study of its potential as an anti-neoplastic agent.

## Conclusions

MASM, derivate of an alkaloid matrine can induce apoptosis and autophagy in cancer cells and possess higher pharmaceutical potency than matrine. Based on signal network analysis it is proposed that the mode-of-action of MASM on cancer cells is regulated by ROS production, and inhibition of autophagy using late-stage autophagic inhibitor CQ increase MASM-induced apoptosis in cancer cells, thus presenting a potential paradigm for exploiting the anticancer properties of MASM. Further studies are necessary to fully understand the mechanism and identify other signaling cascades, which might play a role in observed outcomes. Additionally, in vivo studies need to be carried out to fully ascertain the potential of MASM as a chemotherapeutic agent.

## Supplementary information

**Additional file 1: Figure S1.** LDH release after 24 h of treatment with MASM at various concentrations. **Figure S2.** Flow cytometry scatter plots showing distribution of cells labelled with Annexin V/PI following treatment with MASM at various concentrations clearly showing that post-EMT cells are more susceptible to MASM. **Figure S3.** Effect of pan-caspase inhibitor V-ZAD-FMK on apoptosis induced by MASM in MDA-MB-231 cells. (*p* < 0.01). **Figure S4.** Synergistic effect of MASM and CQ on the viability of MDA-MB-231. (*p* < 0.01). **Figure S5.** Dose-dependent effect of MASM on viability of human pulmonary microvascular endothelial cells.

## Data Availability

All data are part of the manuscript and included in the Figures and Supplementary information and the synthesis of MASM is described in the literature and all cell lines and reagents are commercially available.
