# Supplementary material for: Autophagy inhibition enhances Matrine derivative MASM induced apoptosis in cancer cells via a mechanism involving reactive oxygen species-mediated PI3K/Akt/mTOR and Erk/p38 signaling
Source: BMC Cancer. 2019 Oct 15;19:949. doi: 10.1186/s12885-019-6199-7 (PMC6794878; doi:10.1186/s12885-019-6199-7)
Supplement: Supplementary file 1 — Additional file 1: Figure S1. LDH release after 24 h of treatment with MASM at various concentrations. Figure S2. Flow cytometry scatter plots showing distribution of cells labelled with Annexin V/PI following treatment with MASM at various concentrations clearly showing that post-EMT cells are more susceptible to MASM. Figure S3. Effect of pan-caspase inhibitor V-ZAD-FMK on apoptosis induced by MASM in MDA-MB-231 cells. (p < 0.01). Figure S4. Synergistic effect of MASM and CQ on the viability of MDA-MB-231. (p < 0.01). Figure S5. Dose-dependent effect of MASM on viability of human pulmonary microvascular endothelial cells. [file 12885_2019_6199_MOESM1_ESM.docx]

**Autophagy inhibition enhances Matrine derivative MASM induced apoptosis in cancer cells via a mechanism involving reactive oxygen species-mediated PI3K/Akt/mTOR and Erk/p38 signaling**

Yuming Zou^1,2,3^, Melika Sarem^1,4^, Shengnan Xiang^1^, Honggang Hu^5^, Weidong Xu^3^ and V. Prasad Shastri^1,4,*^

^1^Institute for Macromolecular Chemistry, University of Freiburg, 79104 Freiburg, Germany

^2^Department of Orthopaedics, Department of Orthopaedics, People's Hospital of Wuhan University, Wuhan 430060, Hubei Province, P.R. China.

^3^Department of Orthopaedics, Changhai hospital, Second Military Medical University, Shanghai 200433, P.R. China

^4^BIOSS Centre for Biological Signalling Studies, University of Freiburg, 79104 Freiburg, Germany

^5^Department of Organic Chemistry, School of Pharmacy, Second Military Medical University, Shanghai 200433. P.R. China

*Corresponding author:

V. Prasad Shastri

Email: [prasad.shastri@gmail.com](mailto:prasad.shastri@gmail.com), prasad.shastri@makro.uni-freiburg.de

Stefan-Meier Str. 31

Freiburg, D-79104, Germany

Tel: +49-(0)7612036268

**Supplementary Figures:**


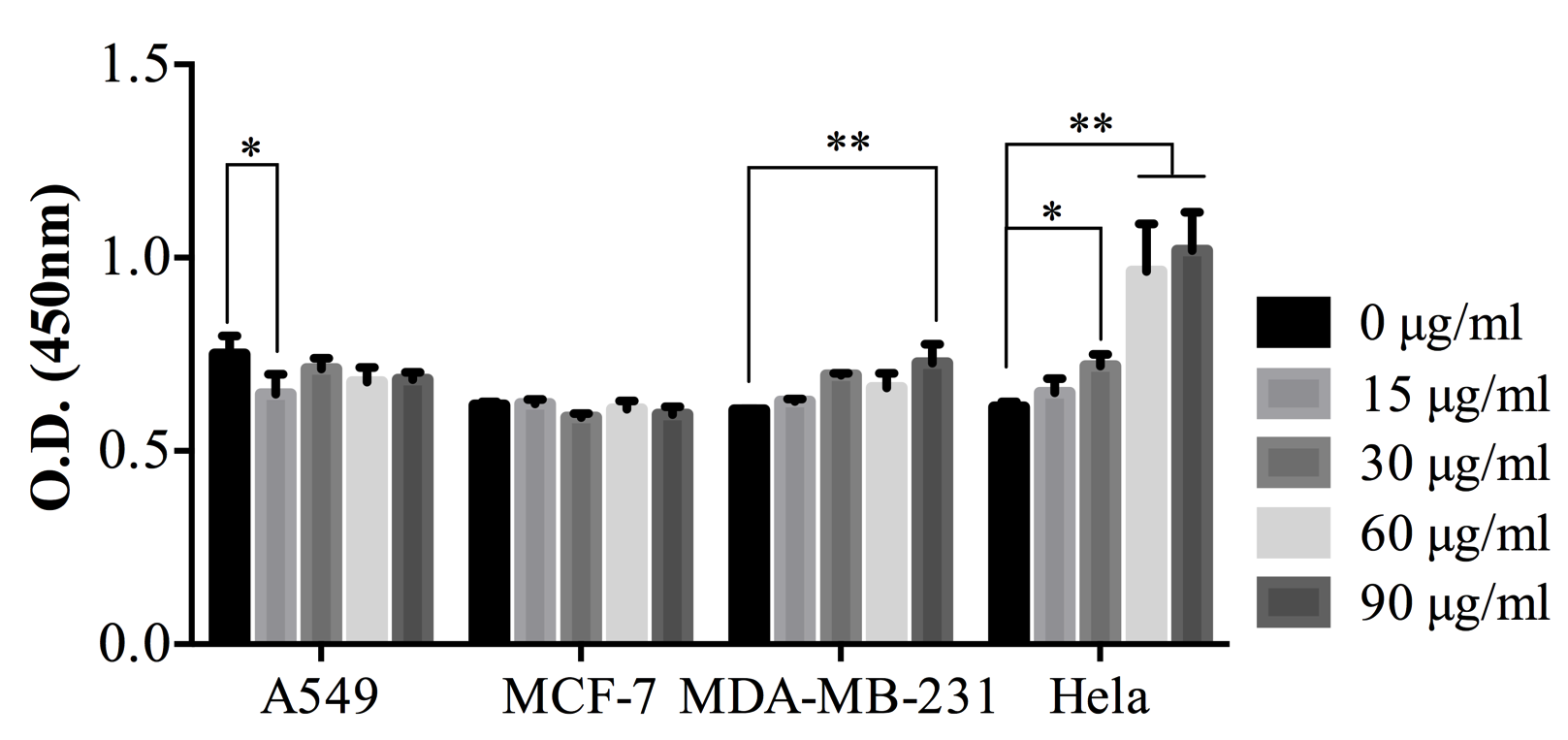


**Figure S1:** LDH release after 24 hours of treatment with MASM at various concentrations.


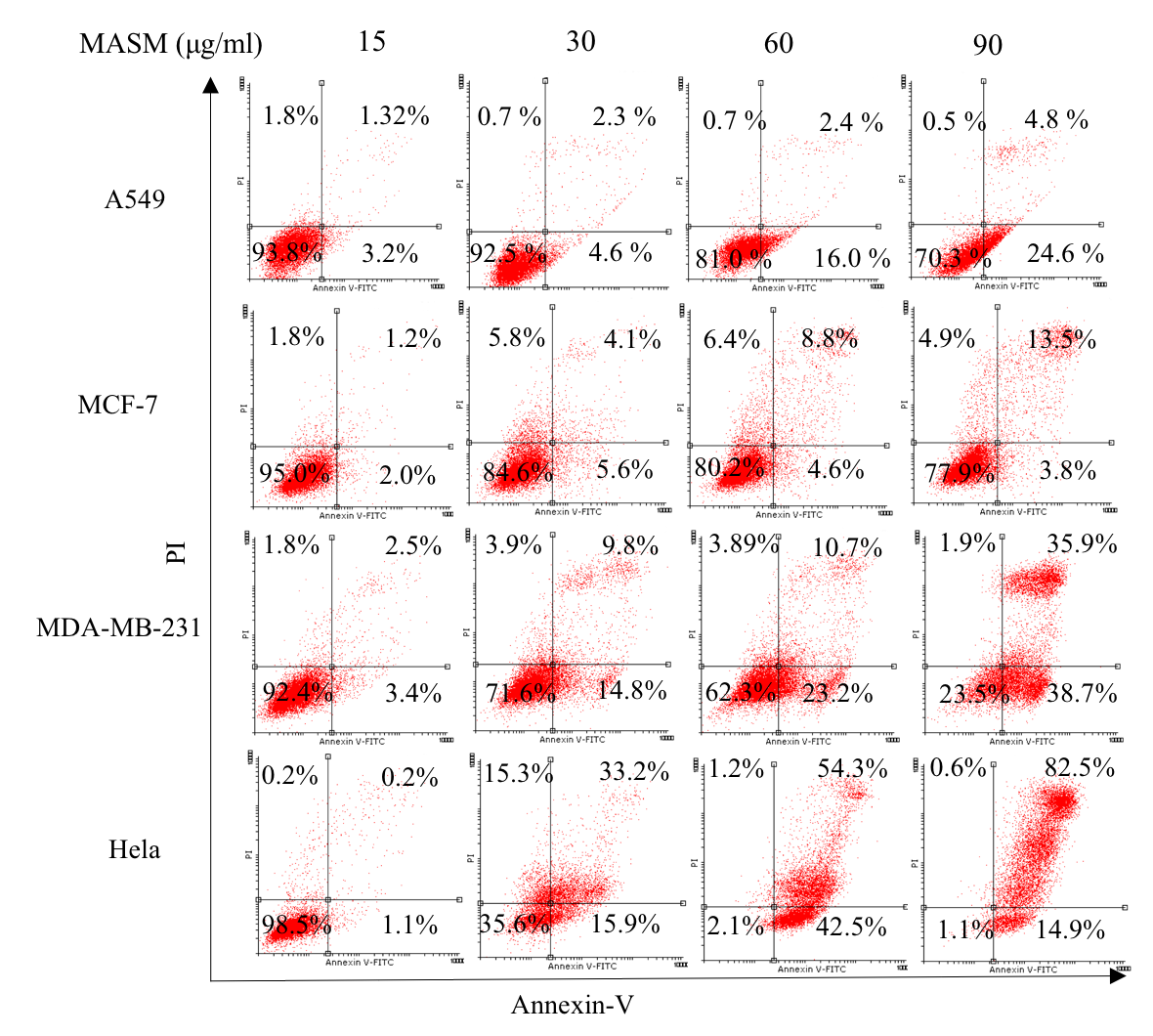


**Figure 2:** Flow cytometry scatter plots showing distribution of cells labelled with Annexin V/PI following treatment with MASM at various concentrations clearly showing that post-EMT cells are more susceptible to MASM.


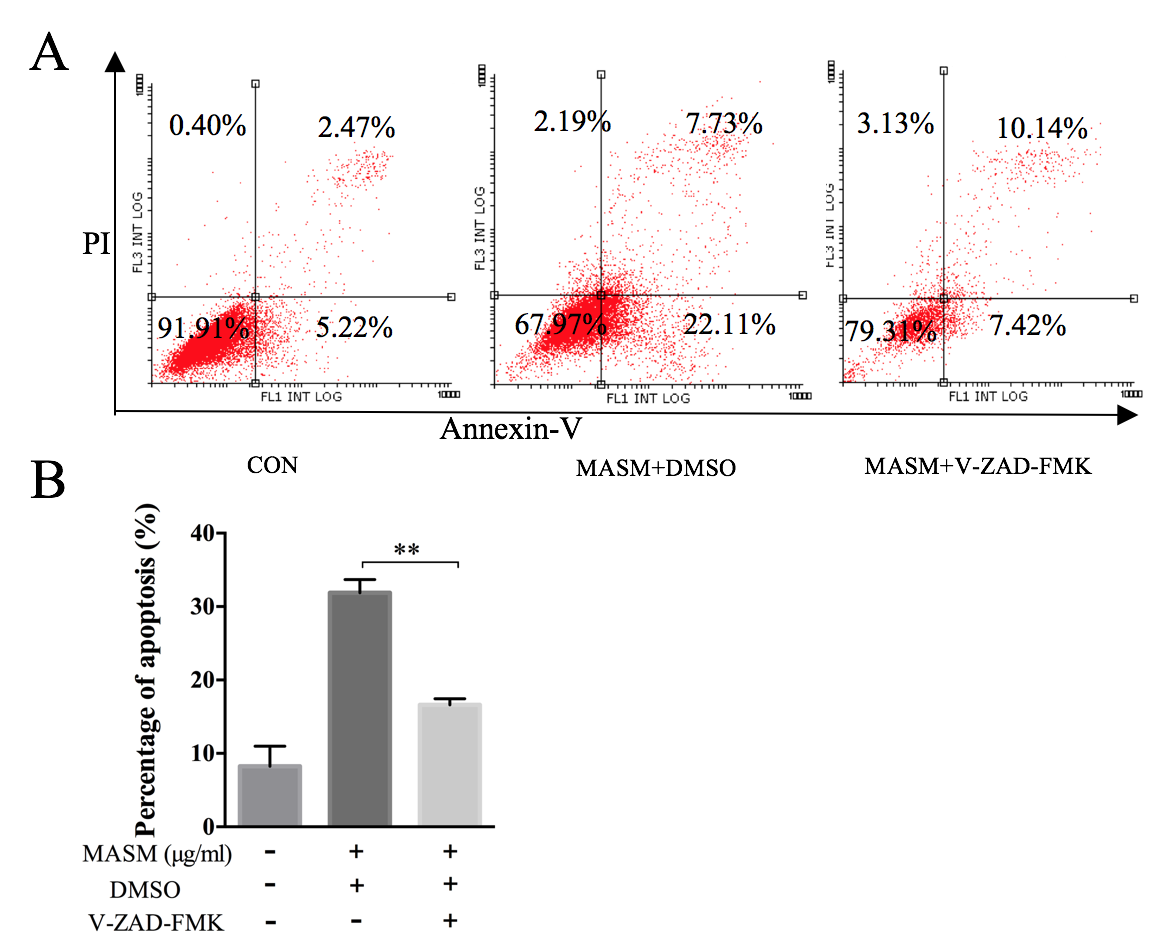


**Figure 3:** Effect of pan-caspase inhibitor V-ZAD-FMK on apoptosis induced by MASM in MDA-MB-231 cells. (p < 0.01)


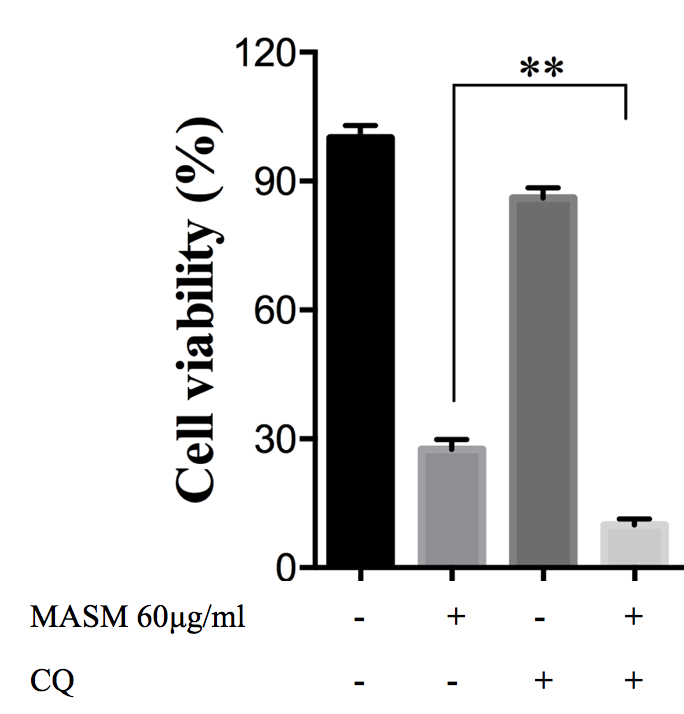


**Figure 4:** Synergistic effect of MASM and CQ on the viability of MDA-MB-231. (p < 0.01)


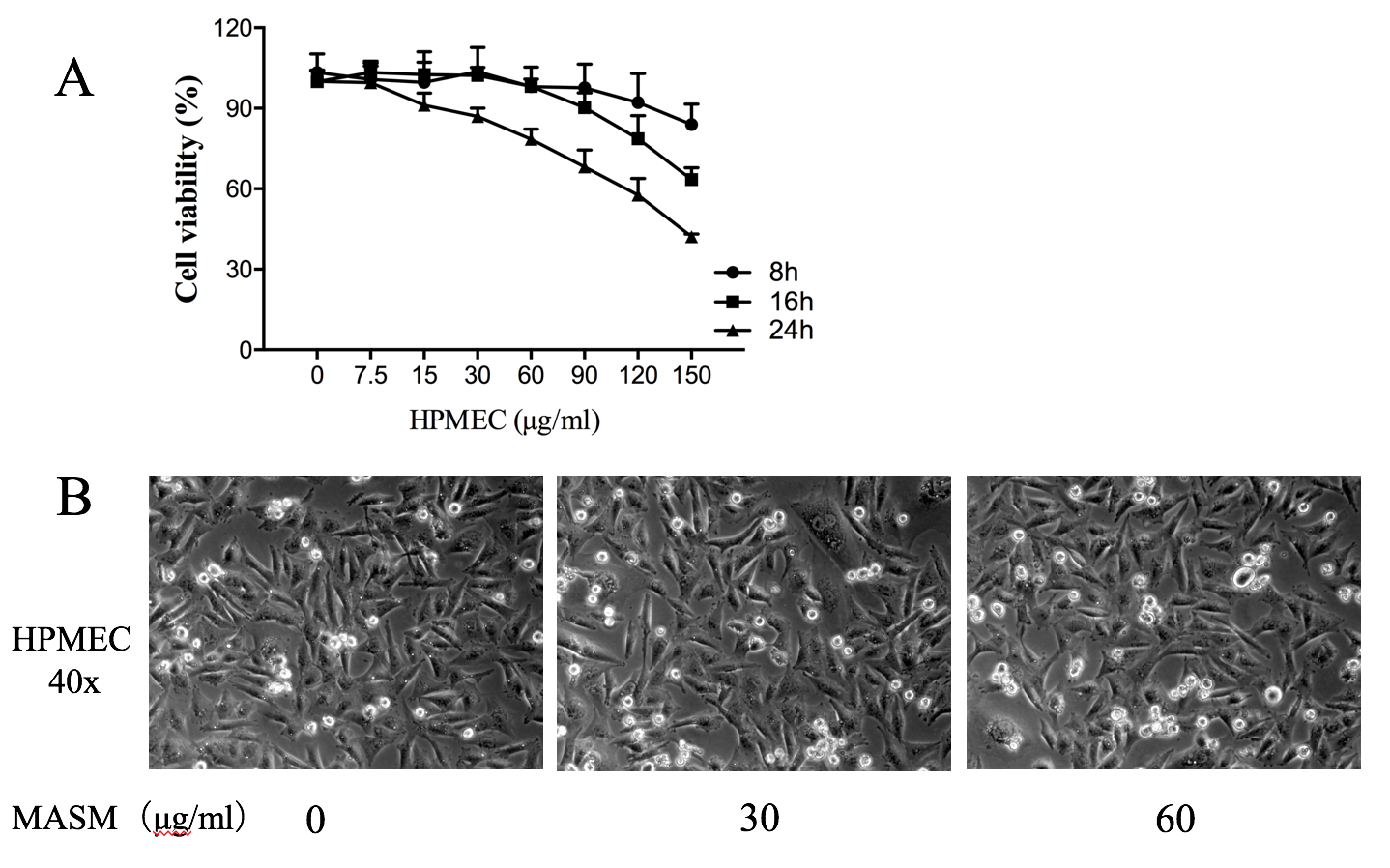


**Figure 5:** Dose-dependent effect of MASM on viability of human pulmonary microvascular endothelial cells.
